# Breeding Schemes: What Are They, How to Formalize Them, and How to Improve Them?

**DOI:** 10.3389/fpls.2021.791859

**Published:** 2022-01-21

**Authors:** Giovanny Covarrubias-Pazaran, Zelalem Gebeyehu, Dorcus Gemenet, Christian Werner, Marlee Labroo, Solomon Sirak, Peter Coaldrake, Ismail Rabbi, Siraj Ismail Kayondo, Elizabeth Parkes, Edward Kanju, Edwige Gaby Nkouaya Mbanjo, Afolabi Agbona, Peter Kulakow, Michael Quinn, Jan Debaene

**Affiliations:** ^1^Excellence in Breeding Platform, Consultative Group on International Agricultural Research, Texcoco, Mexico; ^2^Independent Researcher, Addis Ababa, Ethiopia; ^3^International Maize and Wheat Improvement Center (CIMMYT), Texcoco, Mexico; ^4^International Institute for Tropical Agriculture (IITA), Ibadan, Nigeria

**Keywords:** breeding scheme, breeding pipeline, market segment, product profile, continuous improvement, genetic simulation

## Abstract

Formalized breeding schemes are a key component of breeding program design and a gateway to conducting plant breeding as a quantitative process. Unfortunately, breeding schemes are rarely defined, expressed in a quantifiable format, or stored in a database. Furthermore, the continuous review and improvement of breeding schemes is not routinely conducted in many breeding programs. Given the rapid development of novel breeding methodologies, it is important to adopt a philosophy of continuous improvement regarding breeding scheme design. Here, we discuss terms and definitions that are relevant to formalizing breeding pipelines, market segments and breeding schemes, and we present a software tool, Breeding Pipeline Manager, that can be used to formalize and continuously improve breeding schemes. In addition, we detail the use of continuous improvement methods and tools such as genetic simulation through a case study in the International Institute of Tropical Agriculture (IITA) Cassava east-Africa pipeline. We successfully deploy these tools and methods to optimize the program size as well as allocation of resources to the number of parents used, number of crosses made, and number of progeny produced. We propose a structured approach to improve breeding schemes which will help to sustain the rates of response to selection and help to deliver better products to farmers and consumers.

## Introduction

**FIGURE 1:**
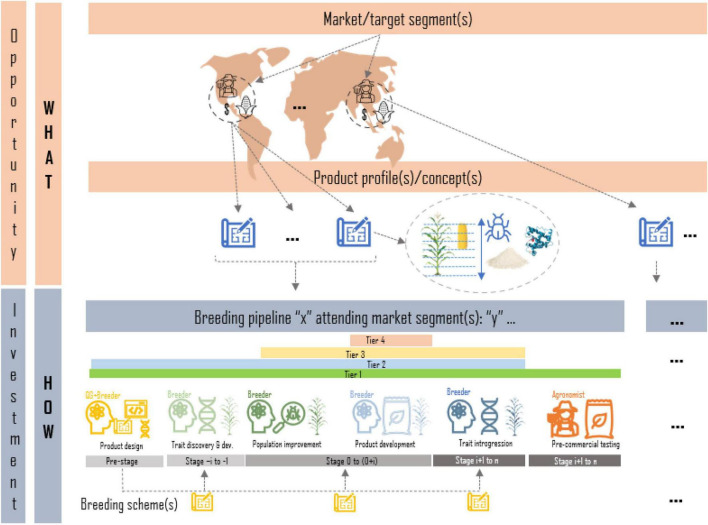
Graphical representation of the relationship between target segments and pipelines of a breeding program. Market segments defined by agroclimatic regions, clients and product features are formalized in product profiles; trait descriptions of desired products to irrupt in the market. Breeding programs are represented as the sum of breeding pipelines focused on one or more of the following tasks: product design, trait discovery, population improvement, product development, trait introgression, or product dissemination. Each breeding pipeline may have one or more breeding schemes (strategies) to attend the market segments and the associated product profiles.

A breeding program is the sum of breeding pipelines to achieve breeding targets for a set of market/target segments^1^ Only after rigorous market and social studies have been carried out and an impactful pipeline investment case is presented to the leadership of an organization/institution, a breeding pipeline is created to carry out trait discovery, population improvement, product development, introgression efforts, seed dissemination/commercialization or a combination of one or several of these (tiers). Any pipeline should have a clear deliverable/product to be handed at the end of the pipeline and a clear customer (another pipeline lead, another organization, etc.). A market segment is defined by the target population of environments in which the final product is grown, as well as descriptions of the target clients and product traits that are valued for production and consumption by farmers and end-users. Products to be placed in a market segment are described through product profiles/concepts; detailed descriptions of the traits and their thresholds (or range of values) to be found in the desired product or variety (sometimes based on current variety in the market) that aims to increase the likelihood of acceptance in the market. A breeding pipeline within a program may target one or more market segments and the associated product profiles using one or more breeding schemes. Breeding schemes are a collection of crossing, evaluation, and selection (CES) tasks and decisions which vary across breeding stages (e.g., in the crossing block vs. advanced yield testing in plants) and ultimately define a breeding strategy (Henryon et al., 2014; Yabe et al., 2017; Cobb et al., 2019; Pook et al., 2020; Gaynor et al., 2021; Figure 1).

Because CES decisions are numerous in a breeding program, breeding schemes can be difficult to describe succinctly and consistently, especially in the context of particular modes of crop reproduction and emerging breeding technologies (Yabe et al., 2017). Breeding leads or other experts typically visualize CES tasks and decisions as illustrative flow charts or tables. Unfortunately, some may not contain all information necessary to reproduce the breeding scheme in other places and may not fully visualize the resource allocation at different stages. Examples of these decisions, which may happen once or repeatedly at different stages of the breeding scheme, are:

• Crossing decisions: number of parents, number of crosses, number of progeny, type of cross, and mate allocation method, etc.
• Evaluation decisions: number of locations, replication level within and among locations, number of checks, experimental design, and plot sizes, etc.
• Selection decisions: percentage of individuals selected (selection intensity), the selection method (e.g., culling, index, tandem), and the selection unit, etc.

**FIGURE 2:**
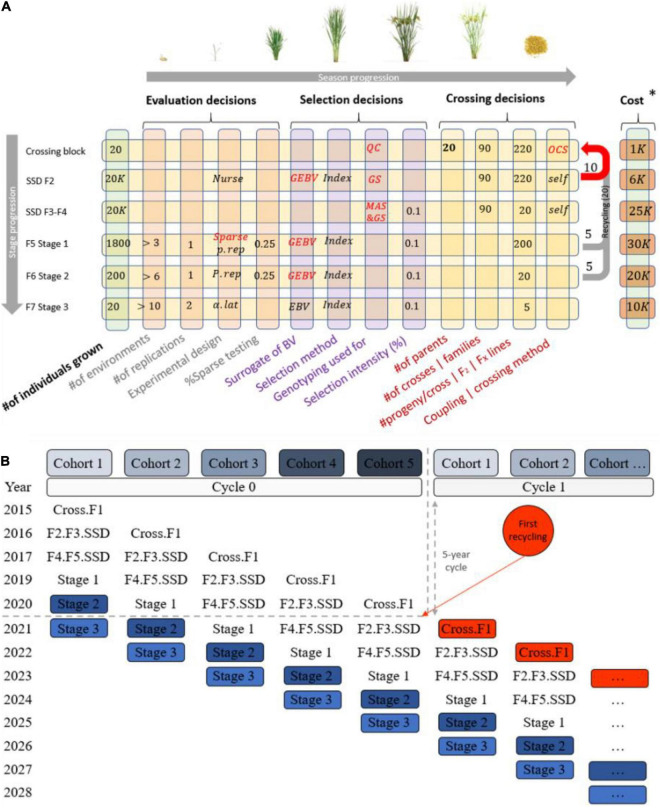
Typical structure of a breeding pipeline. **(A)** Breeding scheme illustrating crossing, evaluation, and selection decisions (columns), which are made once or multiple times across the stage gate process (rows) in a cyclical fashion to achieve genetic gain. **(B)** A graphical representation of cohorts (parallel cycles of breeding) and how they overlap when recycling occurs and parents are taking from multiples stages.

Another layer of complexity in communicating breeding schemes is that the number of stages in a scheme depends on the biology of the species, the multiplication ratio, the evaluation steps required to identify new parents, and the complexity of the market segment and product profile(s) for the desired final product (Henryon et al., 2014). Most breeding programs have a crossing stage to recombine elite parents, stages to multiply progeny and/or generate progeny derivatives such as testcrosses or inbred derivatives (e.g., lines), and multiple stages to test progeny derivatives for their potential as new parents or products. This stage-gate process in breeding programs is repeated cyclically, generating a recurrent selection scheme which, if effective, increases the population mean for the set of traits of interest (Allard, 1999; Chao and Ishii, 2005; Cooper, 2008). Additionally, programs do not wait until a cycle of the stage-gate process is completed to restart the process, and instead run several generations in parallel. A set of genotypes at a given stage within a given cycle is commonly referred to as a cohort or a selection stream (Figure 2). Generations may be discrete or overlapping depending on whether the parents of the cohort genotypes are selected from a single unique cohort or from multiple cohorts. Overlapping generations are more common and lead to more blurred genetic boundaries between cohorts, as cohorts tend to be more related with overlapping generations compared to discrete in absence of inbreeding control (Meuwissen and Sonesson, 1998). In summary, formalized breeding schemes are necessary to clarify the structure of breeding program pipelines.

Despite the inherent complexity of CES decisions, in some organizations breeding schemes are rarely shared formally or presented in writing. It is common for breeding leads to inherit a breeding program and its scheme from their predecessor. Usually, the predecessor transfers the breeding scheme verbally and practically rather than providing a quantitative description of the scheme in a formal document or software. This requires overlap between breeders and on-site presence of the predecessor, potentially for years at a time. Information about the breeding scheme may also be spread among several staff members within the program, interspersed in various publications, or buried in personal notes or presentations. Unfortunately, this method of transferring breeding schemes has led to the total loss of information (and even germplasm) of many breeding programs that have disappeared in the last century (Baenziger, 2006; Gepts and Hancock, 2006; Morris et al., 2006). Improved transferring methods could allow increased interoperability among breeders and better preservation of pedigrees, data, and germplasm.

In addition, codified, systematic documentation of breeding schemes could spur continuous improvement and lead to increased genetic gain and varietal turnover (EiB^2^). As suggested by Bernardo (2002), plant breeding programs should be managed as formal industrial processes that allow better breeding methods to be adopted as they become available to ensure sustainable, steady production of high-quality products. Industrial processes require a clear flow of subprocesses (tasks and decisions) and development of standard operating procedures (SOPs) that ensure minimization of production errors. Several methodologies, such as SixSigma and LEAN among others, were proposed in the 20th century to manage and continuously improve different components of industrial processes in the automotive, communications, and robotics industries (Bhuiyan and Baghel, 2005; Schroeder et al., 2008). Project management tools used in these methodologies, together with modern mathematical and computational tools like simulation and optimization, could easily be extrapolated to draft, formalize, manage, and improve breeding schemes successfully, in contrast to the artisanal approach to breeding common during the 20th century.

Improving a complex process like a breeding program requires understanding of how each process-related decision affects the outcome (e.g., genetic gain or probability of releasing a new product) and how varying these decisions affect the outcome. Given the cost and time associated with piloting new methods or ways to run this complex process, the use of simulations has a particular relevance to the design of breeding schemes (Li et al., 2012; Murray and Atlin, 2017; Yabe et al., 2017). Stochastic simulations of whole breeding programs rarely have been used to improve performance of breeding programs due to lack of computational and software resources in past decades (Gaynor et al., 2021). Currently, simulation technology is available and practical, and it should be incorporated into breeding scheme improvement efforts.

Here, we propose a process to formalize and improve the breeding schemes. In addition, we introduce a publicly available software tool, Breeding Pipeline Manager (BPM), which has capabilities to quantitatively document and record breeding schemes as well as the market segments and product profiles they target in a standardized yet customizable way. The BPM module can be added to any compatible enterprise breeding system (database) to link the phenotypic data to clear targets, pipelines and breeding schemes (Gao et al., 2020). In addition, we discuss the use of classical continuous improvement tools combined with state-of-the-art simulation and mathematical tools to continuously improve breeding schemes. We conclude by providing a case study of the use of these tools and methods in the improvement of the International Institute of Tropical Agriculture (IITA)-cassava breeding scheme. We expect that this framework will assist plant breeding professionals in conducting breeding as a systematic process and to help establish continuity and prevent inconsistency in breeding programs. Furthermore, we expect that formalizing breeding schemes will increase their rates of response to selection (i.e., genetic gain) by motivating critical examination of the schemes used and their opportunities for improvement.

## Materials and Methods

### Enabling Methodology and Software to Formalize Breeding Targets and Schemes

We applied the continuous improvement methodology known as six-sigma to approach breeding as a process and enable breeding scheme improvements. Six-sigma is a five-step method: *define*, *measure*, *analyze*, *improve*, and *control* [DMAIC, (də.′meı.ık)]. The steps are undertaken iteratively to create a cyclical method for continuous improvements. Six-sigma was originally proposed by Bill Smith at Motorola in 1986 to improve industrial processes, in which 99.99966% of all opportunities to produce some features of a part are statistically expected to be free of defects (Tennant, 2017). In the case of plant breeding, this would imply producing better varieties than the ones existing in the market and steady genetic gains with higher probability. The six-sigma method reflects the scientific method, but it is used for process management rather than hypothesis testing. To increase the efficiency and ease of use of six sigma tools (e.g., value stream mapping, correlation analysis, etc.) by breeding teams, we developed a software named Breeding Pipeline Manager (BPM) to document, describe, and visualize market segments, product profiles and breeding schemes. BPM is available at http://bpm.excellenceinbreeding.org? In collaboration with multiple breeding programs for a wide range of crops within the CGIAR, such as line, hybrid, and clonally propagated species, we identified breeding decisions which fell into three categories: crossing, evaluation and selection decisions. We then summarized breeding schemes and decisions as a table containing the breeding stages in rows (e.g., seedling nursery, stage 1 yield testing, etc.) and the CES tasks and decisions in columns. BPM provides a graphical user interface for capturing breeding schemes in a standard format. BPM also allows users to create visualizations (flowcharts) of their breeding scheme. In addition, the BPM allows market segment and product profile definition and users can link breeding schemes to market segments.

The BPM back-end was developed in Node JS, an open-source, cross-platform, and scalable JavaScript runtime environment. The front-end graphical user interface was developed in React JS. The source code is available at https://gitlab.com/excellenceinbreeding/module2. The platform leverages NodeJS asynchronous technology to perform intensive calculations without affecting the performance of other functionalities of the system. In addition, the platform uses Docker containerization technology for continuous development and integration (Merkel, 2014; Boettiger, 2015). This will not only enable automation of the deployment process but also horizontal scaling on any cloud infrastructure (depending on traffic). An online manual showing the details of the available features of the software and their use can be found once connected in the tool under the question mark bar at the bottom menu.

### Application of Continuous Improvement in Breeding Programs: IITA-Cassava Example

We selected the CGIAR IITA-Cassava program to showcase the importance of using enabling tools and simulations to continuously optimize breeding programs. The IITA cassava program is situated in different parts of sub-Saharan Africa to serve region-specific challenges and market segments. The IITA-Cassava east-Africa pipeline, situated in Uganda, was chosen to showcase the use of SixSigma and the BPM tool to improve their breeding scheme. The five six-sigma steps were applied as follows in the cassava program. The *problem was defined* as lower than achievable genetic gain for traits of interest under the current scheme. The breeding targets and scheme were *measured* (documented) by capturing all CES decisions across all stages using the BPM tool (as described in the next section) through several interactions with the breeders. The analysis of the measured decisions and the genetic gain indicators revealed many possible improvements. We first chose to *analyze* the crossing decisions in the breeding scheme to identify possible improvements. The number of parents (nParents), number of crosses (nCrosses), number of progeny per cross (nProgeny), and recycling strategy were prioritized for evaluation *via* stochastic simulation in AlphaSimR (Gaynor et al., 2021). We proposed an *improvement* plan based on the close-to-optimal number of parents, number of crosses, number of progeny, and recycling strategy identified *via* simulation. The improvement plan used the A3 format (referring to the size of an A3 sheet that describes a project briefly) common in project management (Anderson et al., 2011). We then *controlled* the improvement by monitoring how key performance indicators (a set of quantifiable measurements used to gauge an institution’s overall long-term performance) stated in the improvement plan changed as the improvements proceeded.

### Stochastic Simulation to Improve Crossing Decisions in IITA-Cassava East-Africa Pipeline

#### Current and Alternative Programs

As a clonally propagated crop, cassava breeders currently have adopted a four-stage evaluation strategy in addition to the crossing block stage and the seedling nursery stage where planting material is multiplied. These evaluation stages include stage 1 (clonal evaluation; CE), stage 2 (preliminary yield trial; PYT), stage 3 (advanced yield trial; AYT), and stage 4 (uniform yield trial; UYT; Table 1). The summary of the advancement decisions across the different stages in the current (baseline) pipeline that was to be improved is as shown in Table 1. The pipeline began with only four parents selected to have the target traits for the target markets. From the four parents, 12 crosses were made, each with 136 progeny, thus resulting in 1,632 individuals. All 1,632 were multiplied in the seedling nursery and then evaluated at stage 1 in one environment and one replication per environment. Based on performance at stage 1 testing, 120 individuals were selected and advanced to stage 2 testing in two environments and two replications per environment. From stage 2 evaluation, 64 individuals were selected and advanced to stage 3 testing in two environments and three replications per environment. Finally, 24 individuals were selected and advanced to stage 4 testing in two environments and three replications per environment. Recycling of parents was planned to occur at PYT and UYT. This information was input into BPM and the scheme was simulated to address specific questions related to crossing decisions as prioritized by the breeding team. The program was interested in knowing if the use of four parents was adequate to sustain genetic gain. The program also inquired how to improve their recycling strategy, particularly from which stage to recycle and whether to recycle from multiple stages. Here, we share the results for improving these decisions among many others that are currently being improved. It should be noted that the IITA pipelines in other regions, particularly for West Africa, use a greater number of parents (∼100) in their crossing block and therefore were not subject to this improvement. The simulation exercise is expected to find an optimal number of parents between these two extremes and useful for the East-Africa pipeline and develop some high-level guidelines for the test of the IITA-cassava pipelines.

**TABLE 1:** Summary of IITA-Cassava east-Africa pipeline numbers handled by stage.

Stage	Year	nParents	nCrosses	nProgeny/ cross	nIndividuals	% Selected
Crossing block	1	4	12	136	1,632	–
Seedling nursery	1	–	12	136	1,632	100
Stage 1 (CE)	2	–	12	136	1,632	100
Stage 2 (PYT)	3	–	–	–	120	7.35*
Stage 3 (AYT)	4	–	–	–	64	53.3*
Stage 4 (UYT)	5-6	–	–	–	24	37.5

*Stages where the recycling occurs to form the new crossing block. Recycling from the combined PYT and AYT leads to an average cycle time of 3.5 years.

### Simulation Parameters: Treatments

To keep the resources constant with the baseline, we restricted the number of individuals (nIndividuals) at the F1 stage to 1,632 in all experimental simulation treatments. We then developed a grid to evaluate different numbers of parents in the crossing block using the following possible numbers of parents (nParents): 4, 8, 16, 32, and 64. The number of possible crosses for each level of number of parents was constrained to a maximum of nParents * (nParents − 1)/2, which is equivalent to all possible combinations of parents or a half-diallel, while considering the initial restriction that the number of individual progeny (nIndividuals) must be equal to 1,632. This resulted in the following possible numbers of crosses: 6, 12, 24, 48, 96, 204, 408, or 816. To keep the number of individual progeny constant at 1,632, the number of progeny per cross was set to 272, 136, 68, 34, 17, 8, 4, 2 for numbers of crosses equal to 6, 12, 24, 48, 96, 204, 408, and 816 respectively. The number of individual progeny is always equal to the number of crosses multiplied by the number of progeny per cross.

As such, a total of 24 simulation treatments were defined (Table 2). To identify the optimal number of parents, number of crosses, number of progeny per cross, and the best recycling strategy, a stochastic genetic simulation was conducted in the R package AlphaSimR (Gaynor et al., 2021).

**TABLE 2:** Summary of factor values combined for number of parents, number of crosses, and number of progeny per cross to produce a total of 1,632 progeny.

Number of Parents	Number of Crosses	Number of Progeny per cross
4	6	2
8	12	4
16	24	8
…*	…*	…*
64	816	272

…* indicates the numbers duplicate until reaching the final numbers in the row. All treatment combinations going beyond the 1,632 progeny were not run. This allowed comparison of these factors’ influence on genetic gain at a fixed program size.

### Simulation Parameters: Genome and Evaluation

#### Burn-In Genome Sequence

For each replicate, a genome consisting of 18 chromosome pairs was simulated for the hypothetical plant species similar to cassava. These chromosomes were assigned a genetic length of 1.43 Morgans and a physical length of 8 × 10^8^ base pairs. Sequences for each chromosome were generated using the Markovian coalescent simulator (MaCS; Chen et al., 2009) implemented in AlphaSimR (). Recombination rate was inferred from genome size (i.e., 1.43 Morgans/ 8 × 10^8^ base pairs = 1.8 × 10^–9^ per base pair), and mutation rate was set to 2 × 10^–9^ per base pair. Effective population size was set to 30 to mimic an evolution history of natural and artificial selection.

#### Burn-In Founder Genotypes

Simulated genome sequences were used to produce 4 founder non-inbred individuals. These founder individuals served as the initial parents in the burn-in phase. Sites segregating in the founders’ sequences were randomly selected to serve as 100 quantitative trait nucleotides (QTN) per chromosome (1,800 total).

#### Burn-In Phenotypes

A single highly complex trait representing an index of tuber yield, dry matter, cassava mosaic disease, total carotenoids and sprouting was simulated for all founders. The genetic value of this trait was determined by summing its QTN allelic effects. To model genotype-by-environment (GxE) interaction, allele effects depends on the value of an environmental effect which changes over years. For a given year, the allele effects followed this formula:


$$a_{i}\,\left(w_{j}\right)=b_{i}+m_{i}\,w_{j},$$

where *a*_*i*_ is the allele effect for QTN *i*, *w*_*j*_ is the environmental effect for year *j*, *b*_*i*_ is the QTN intercept and *m*_*i*_ is the QTN slope on the environmental effect. The slope, intercept, and environmental effects were sampled from the following normal distributions. This equation is equivalent to Finlay–Wilkinson regression. Details on the full formulation of genotype by environment interaction simulation features enabled in AlphaSimR can be found in Gaynor (2021). In the case of the cassava program, a variance component for genotype by year ($\sigma_{G\,x\,Y}^{2}\,2$) and genotype by location ($\sigma_{G\,x\,L}^{2}\,1$) interactions were defined and summed to produce the genotype by year by location ($\sigma_{G\,x\,Y\,x\,L}^{2}\,3$) interaction variance components used in the addTraitAG() function in the varGxE argument in AlphaSimR for a trait with additive gene action and GxE interaction. Main genotype variance component was assumed equal to 1 (σ1G2). The genetic values of each non-inbred individual were used to produce phenotypic values by adding random noise sampled from a normal distribution with mean 0. The variance of the random error was varied according to the three stages of evaluation in the breeding program based on the plot size and number of replications per entry currently used according to the different experimental designs used at the different stages (augmented design at stage 1 and randomized completely blocked design in posterior stages). The values for these error variances were set to achieve levels of plot heritability reported by the cassava program currently estimated at the different stages.

In order to simulate the multi-environment testing common in breeding programs, the variance components for genotype by year and genotype by locations were used to simulate a matrix of possible slopes for the environmental covariate used by the setPheno() function in the *p*-value argument (years in rows and locations in columns). The values were sampled depending on the year and number of locations used for a given stage to approximate the GxE. A summary of simulation features for the genome and phenotypes can be found in Table 3.

**TABLE 3:** Summary of simulation features for the genome and phenotypes.

Simulation features		
Burn-in	Genome sequence	100,000 generations of evolution
		18 chromosome pairs
		1.43 Morgans per Chromosome
		8 × 10^8^ base pairs per chromosome
		2 × 10^–9^ mutation rate
	Founder genotypes	4 non-inbred founders
		1,800 QTN (additive GxE effects)
		Normally distributed QTN effects $\sigma_{G\,x\,Y}^{2}\,2$, $\sigma_{G\,x\,L}^{2}\,1$, $\sigma_{G\,x\,Y\,x\,L}^{2}\,3$, $\sigma_{G}^{2}\,1$
	Recent breeding	20 years of modern breeding
		Non-inbred cloned individuals
		Conventional breeding
Evaluation	Future breeding	20–60 years of breeding
		Testing alternative allocation of resources
		Equal cost programs
		Conventional breeding

Population means and standard errors at Stage 1 of yield evaluation across the 20–60 years of breeding for the treatments described previously were computed using the dplyr library in R (Wickham et al., 2021), and plotted using the ggplot2 library in R (Wickham, 2011). One hundred replicates were run for each simulation treatment.

## Results and Discussion

### Adapting Continuous Improvement Tools and Concepts in the Improvement of Breeding Schemes

**FIGURE 3:**
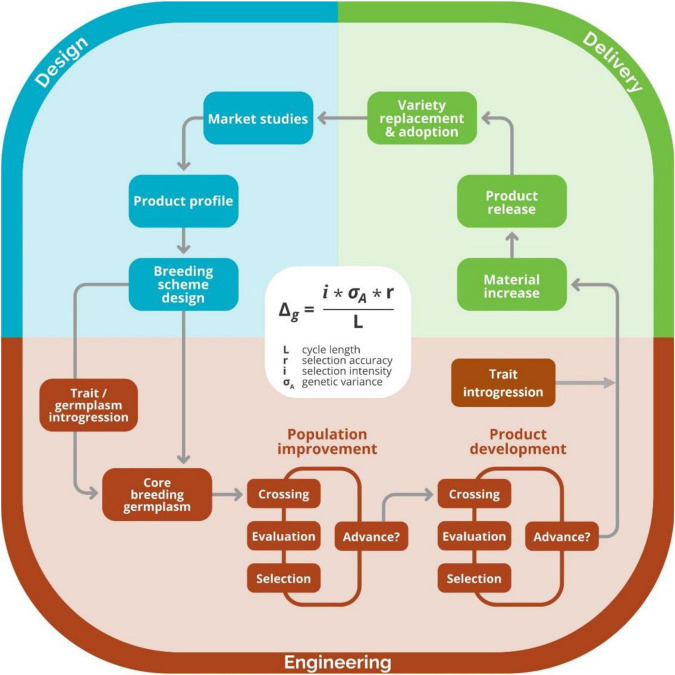
Graphical representation of breeding as a process. The design component of the breeding process, which includes activities such as defining market segments, product profiles, and breeding schemes, is shown in blue. The engineering component of the process, where crossing, evaluation, and selection activities for product development and population improvement are made, is shown in red. The delivery component of the breeding process, where activities like material increase and registration, occur are shown in green. Image taken with permission from Covarrubias-Pazaran (2020).

**FIGURE 4:**
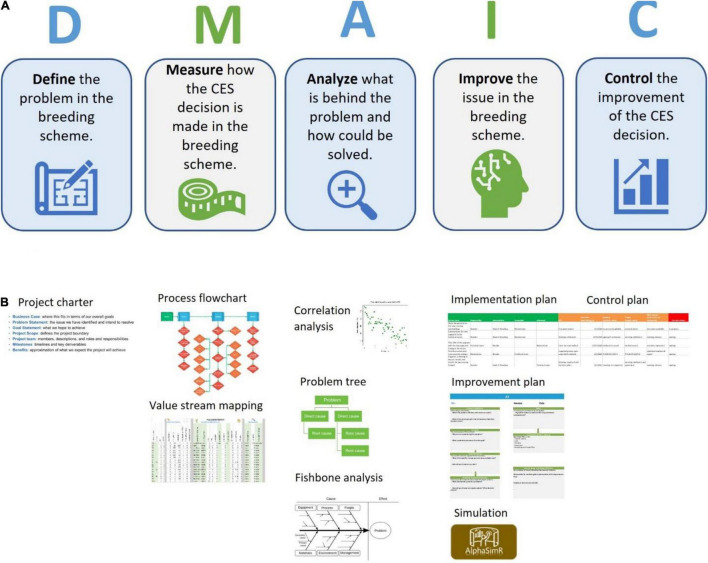
Graphical representation of the six-sigma process applied to the continuous improvement of breeding schemes (strategies). **(A)** Description of the DMAIC steps. **(B)** Different tools to support continuous improvement of crossing, evaluation, and selection (CES) decisions in breeding schemes.

Following the paradigm of approaching breeding as an industrial process (Bernardo, 2002), we adapted the six-sigma methodology to continuously improve the breeding schemes of breeding programs (Figure 3). Under this framework, we follow the project methodology Plan-Do-Study-Act inspired by William Edwards Deming named DMAIC, an acronym standing for Define, Measure, Analyze, Improve and Control steps that are cyclically repeated to reflect the continuous or cyclical approach (Aguayo, 1991; Figure 4). To demonstrate the use of the continuous improvement methodologies to optimize breeding processes leveraging from measuring tools like the breeding pipeline manager (BPM) and stochastic simulation, we engaged in discussions with the IITA Cassava program. First, the cassava team registered their breeding pipelines and the market segments targeted per pipeline. We found the IITA-cassava program to be composed of five breeding pipelines and on average tackling six market segments. The market segments and accompanying pipelines are stratified by a combination of regional consumption preferences and prevailing biotic and abiotic stresses. For example, most of the produced cassava in West Africa goes to processed (granulated and paste) products while in east Africa, the predominant preference is for fresh consumption with minimal processing (boiling, roasting and flour from dried roots). Subsequently, we focused on the IITA-Cassava east-Africa pipeline targeting market segments listed by breeders and generally described as fresh market and high-quality flour. Even though we proposed six-sigma for improving breeding schemes, the reader should keep in mind that continuous improvement applies to all components of the breeding process including the management roles which are responsible of the encouraging and incentivizing improvements.

### Defining a Problem

The step of *defining* the problem was adapted to breeding scheme improvement by defining the problem as a suboptimal rate of response to selection (genetic gain) but pointing to one of the many crossing-evaluation-selection (CES) tasks and decisions at a given stage as the possible root of the problem. We found tools such as Project charter useful to define the problem (McKeever, 2006). We proceeded to use the project charter to *define* or state the problem in the IITA-Cassava east-Africa pipeline as having “potential for greater response to selection without increased expenditures.” Details in the business case, goal statement, timeline, scope, and team members can be found in Table 4. Unfortunately, we found that estimates of realized genetic gain were not available in the program to justify the definition of the problem. However, given the lack of an efficient recurrent selection strategy, we assumed the definition of the problem to be relevant to the program.

**TABLE 4:** Project charter applied to the IITA-Cassava east Africa program.

**Problem statement** The rate of genetic gain in the IITA-Cassava east-Africa breeding program is less than or equal to 1% per year for traits of interest, and the rate of variety turnover is lower than possible.	**Business case** By optimizing the breeding schemes using quantitative genetics principles, we can increase the response to selection per dollar invested per unit time.

**Goal statement** Reduce cycle time to the biological limit, optimize the trade-off between selection intensity and accuracy, manage the genetic variance, while constraining possible alternatives to similar level of resources.	**Team members** Cassava head of breeding Cassava breeders Quantitative Geneticist
**Scope** Crossing, evaluation and selection (CES) decisions included in the breeding scheme.	**Timeline** One to two CES tasks and decisions improved per year.

### Software Development to Measure/Document Breeding Programs

**FIGURE 5:**
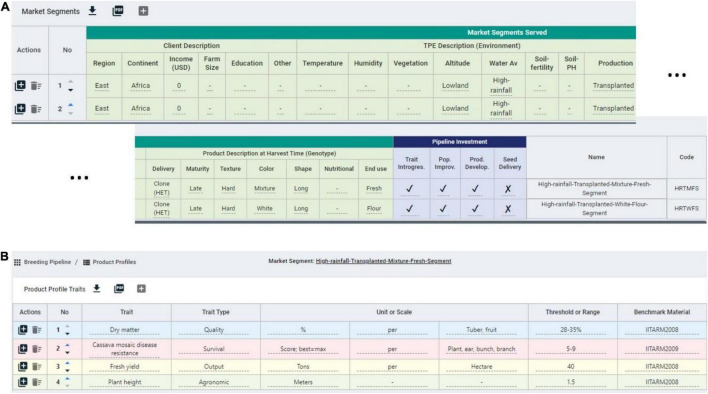
Snapshot of some market segments and product profiles defined in the breeding pipeline manager (BPM) tool for the IITA-cassava east-Africa pipeline. **(A)** Two market segments for a cassava program with defined client, environment and product features, mainly distinguished by the end use. **(B)** Example product profile for a cassava market segment featuring quality, survival, output, and agronomic traits.

To facilitate the *measuring* step of the continuous improvement approach proposed, in which CES decisions are recorded for further *analysis* (Bhuiyan and Baghel, 2005), we developed the BPM software. The breeding pipeline manager tool (BPM) is equipped with a module to define breeding pipelines as the sum of efforts to deliver a product. Breeding pipeline definition is the highest-level unit of information clustering in the BPM tool. The pipeline can be linked to market segments defined by the user. The market segment is defined in the BPM tool as the sum of the client, the target population of environments (TPE), and final product characteristics displayed in Table 5. These aim to capture the characteristics that can make breeding a more targeted effort according to Ragot et al. (2018) (Figure 5A). The BPM module can be incorporated to any enterprise breeding system (database) to properly link the generated phenotypic data to clear target segments and pipelines. Market segments for the cassava pipeline were captured using the BPM tool and are shown in Figure 5A. The major focus is on lowland high-rainfall, late maturity, long, hard cassava for fresh and flour consumption.

**TABLE 5:** Features defining a market segment.

Client features	Environment features	Product features
Geographical region	Temperature	Mode of reproduction
Income	Humidity	Maturity
Education	Vegetation	Color
Farm size	Water availability	Shape
	Soil fertility	Biofortification
	Altitude	End use
	Soil pH	
	Production system	
	Prevailing biotic stresses	

*The features of the client being served, the features of the target population of environments (TPE), and the final product characteristics are displayed. These three sets of features define a market segment in the breeding pipeline manager (BPM) tool.*

On top of defining the market segments, breeding programs must describe specifics of the product to be released in the market. Here, the concept of product profile (sometimes referred to as product concept) applies (Ragot et al., 2018; Carey et al., 2021). The existence of these profiles can make the difference between success and failure (Carey et al., 2021; Mwanga et al., 2021). The BPM tool has a module to define product profiles and link them to specific market segments, and the cassava breeders used the tool to formalize such profiles (Figure 5B). One of the product profiles for example is focused on achieving defined levels of fresh yield, plant height, dry matter and cassava mosaic disease resistance.

Part of the design of breeding pipelines is the creation of a blueprint or a breeding scheme that will allow the breeder to achieve the product profile for the market segment while maximizing the genetic gain of the breeding population per dollar invested (Henryon et al., 2014). The *blueprint* should specify all the crossing, evaluation and selection tasks and decisions occurring at the different stages (e.g., recombination, multiplication and testing stages) for the purposes of population improvement and product development. Most breeding programs have these two purposes coupled in a way that advancement decisions influence the recycling decisions. Others have proposed and shown that decoupling the population improvement from product development by moving the recycling decision to very early stages (e.g., F2, nursery or multiplication stages) will increase the rates of genetic gain. Better products can be expected when the product development process is regarded as separate from a rapid cycling population improvement strategy (Gaynor et al., 2017).

Crossing, evaluation and selection (CES) decisions comprising the breeding scheme can and should be recorded at the highest level of detail and safeguarded for the benefit of the breeding organization in case of any adverse circumstances. In Table 6 we show the CES decisions that should be considered to capture the level of resolution and detail necessary to avoid loss of valuable information; these can be recorded by the BPM tool in the breeding scheme module. The software allows for breeding pipelines to manage multiple breeding schemes, as may happen when a program has a principal breeding scheme, but one or more parallel experimental breeding schemes, to accelerate genetic gains.

**TABLE 6:** Examples of crossing, evaluation and selection decision recorded by the BPM tool across the different stages of the breeding program, defining the breeding strategy.

Evaluation	Selection	Crossing
Plant portion harvested in the previous season to be planted in the current season (e.g., seed, tuber, cutting)	Surrogate of merit (e.g., BLUE, BLUP, GBLUP) per phenotyped trait	Crossing or multiplication unit (e.g., family, individual)
Cultivation method of the plant portion (e.g., pot, plot, petri dish)	Number of locations per phenotyped trait	Crossing or multiplication method (e.g., 2-way cross, 3-way cross)
Experimental design	Selection method (e.g., visual, culling, index)	Parent coupling method (e.g. random mating, optimum contribution)
Total number of locations	Method to model genotype x environment interaction	Number of potential female parents
Replications per location	Method to model spatial adjustment	Number of potential male parents
Plot width and units (e.g., 1 m^2^)	Selection intensities for different selection units (e.g., families, lines, female parents)	Total number of crosses or total number of unique materials to multiply
Plot length and units (e.g., 1 m^2^)	Recycling unit	Number of progeny per cross or number of clones multiplied
Sparse testing percentage	Recycling generation	Molecular technology
Sparse testing bridging method	Number of selection units recycled	Number of molecular marker sites
Number of checks		Purpose of molecular technology (QC, GS, etc.)
Percentage of check plots		Population used in genomic selection as the training (prediction) set

### Measuring the Process

**FIGURE 6:**
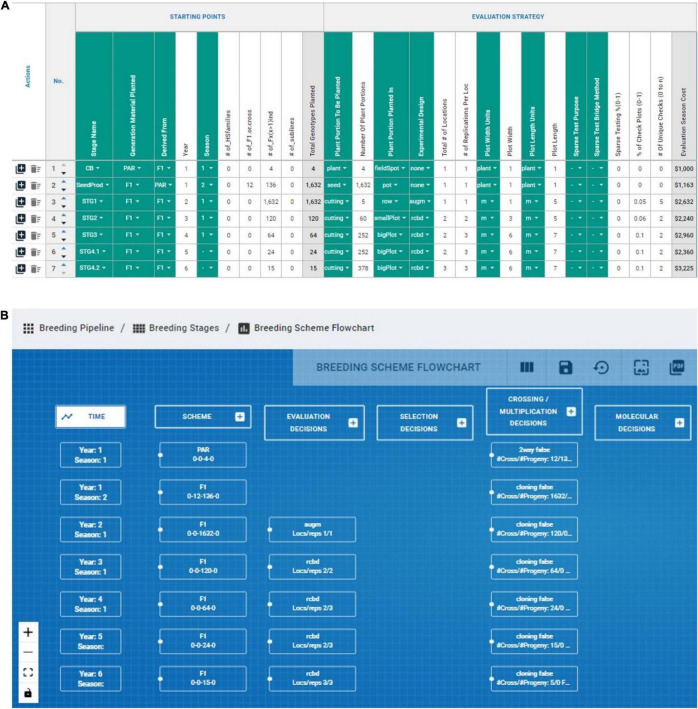
Graphical representation of BPM capabilities to record and display breeding schemes. **(A)** The evaluation decision across stages of an IITA-Cassava breeding scheme mapped in the breeding pipeline manager (BPM) tool are displayed in columns, and sequential stages are displayed in rows. **(B)** The capability to draw flowcharts with the available information in the breeding schemes is displayed.

The *measuring* step of the six-sigma process was adapted by recording numerically and categorically all the different CES decisions across the various stages directly impacting genetic gain (e.g., number of parents, # crosses, # progeny, coupling method, etc.). The lack of available tools to measure/record breeding schemes was the motivation to develop the BPM tool presented above, although the BPM tool is inspired by the Value Stream Mapping approaches commonly used in process management (Singh et al., 2011). We *measured* or recorded the breeding scheme of the IITA-Cassava program using the BPM tool to capture all crossing, evaluation and selection decisions across the different breeding stages and a portion can be observed in Figure 6. We captured seven stages (crossing block, multiplication and five stages of yield and agronomic evaluation) across 52 different CES decisions for the East-Africa cassava pipeline that informed the *analysis* step to identify areas for improvement (Figures 4, 5). These decisions comprise the crossing, evaluation and selection strategies for both population improvement and product development.

### Analyzing the Problem

The *analysis* step was adapted to breeding scheme improvement by replacing approaches such as correlation analysis. Correlation analysis is a method that links a response variable or key performance indicator (KPI) to another variable in the production process to understand relationships that could indicate the part of the process that needs to be refined. We instead conducted an analysis based on known quantitative genetic relationships between the various CES decisions and genetic gain (e.g., program size affects genetic gain depending on how effectively genetic variance is utilized and also linked to selection intensity). Additional tools like Fishbone (diagram to articulate the root causes of the problem) are not discouraged but we limited this exercise to one-to-one meetings with the breeding team to discuss the possible gaps while analyzing the current scheme together in the light of quantitative genetic principles (Ishii and Lee, 1996). We initially found several possible areas of improvement, including the small size of the program, the experimental design used at yield and agronomic evaluation stages, the coverage of the target population of environments (TPE), the opportunity to use molecular information, the potential improvement of analytical methods for genetic evaluation, a possibility to select the best families at earlier stages, the possibility to reduce the cycle length, and other decisions such as an improved crossing plan. Since it is well-known from classic quantitative genetics theory that using resources properly to maximize the genetic variance observed among and within families can maximize response to selection (Lynch and Walsh, 1998; Hallauer et al., 2010), we chose to optimize the decisions of number of parents, number of crosses and number of progeny per cross given the low number of parents used by the program in the crossing block and very likely limiting the rate or sustainability of genetic gains. Although we first focused on improving the resource allocation for the number of parents, crosses and progeny, the reader should remember that as a continuous improvement process, the other areas of opportunity identified should also be improved right after or at the same time depending on the resources available. This is just an example of how to implement breeding scheme improvement.

### Using Simulation to Optimize the Process

**FIGURE 7:**
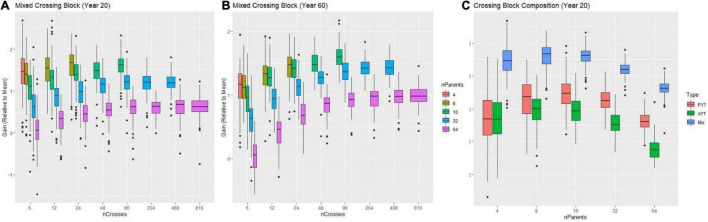
Results from simulations comparing different numbers of parents and crosses combinations subject to the constraint of ∼1,600 individuals manageable for a time-horizon of 20 and 60 years of breeding. In panels **(A,B)** the genetic gain (relative to the mean) measured in advanced yield trial (AYT) individuals is shown (y-axis) as a function of a different number of crosses (x-axis) for two breeding-time horizons (20 and 60 years) for different number of parents (colored boxes) is shown. In panel **(C)** the genetic gain (relative to the mean) measured in advanced yield trial (AYT) individuals is shown (y-axis) as a function of a different number of parents (x-axis) for the breeding-time horizon of 20 years comparing different compositions of the crossing block are shown. The red, green and blue boxes represent crossing blocks composed by recycling AYT, PYT, or a mix of PYT and AYT individuals respectively.

Prior to recommending an *improvement* plan, we used genetic simulation (Gaynor et al., 2021) to identify optimal use of resources (plots available) by defining a grid of possible treatments that contained different combinations of number of parents, crosses and progeny subject to the constraint of 1,632 individuals at the F1 stage assuming other factors constant (e.g., properly resourced, properly tested, etc.). Regarding recycling strategy, using overlapping cohorts to recycle (i.e., a mixed crossing block composed half of parents from the PYT and half of parents from the AYT) lead to higher genetic gain regardless of the number of parents (Figure 7A). Based on this observation, we evaluated the effect on genetic gain of the number of parents, crosses and progeny while recycling from the mixed PYT and AYT.

For the single complex trait which represented an index of multiple traits, the decision of the number of parents provided the greatest opportunity to increase genetic gain. An excessive number of parents -here, more than 30- always resulted in decreased genetic gain compared to use of fewer than 30 parents at both the 20 and 60-year time horizons (Figure 7C). At the 20-year time horizon, the optimal number of parents was ∼8–16. However, at the 60-year time, the optimal number of parents increased to between 16 and 32.

Increasing the number of crosses generally increased gain, but with diminishing returns to additional crosses at a given number of parents. At low numbers of parents, not enough possible unique crosses were available to take advantage of gains possible by increasing the number of crosses. Interestingly, the optimal number of crosses also differed in the short (20-year) and long (60-year) terms. At the 20-year timepoint, schemes with fewer crosses and more progeny per cross tended to have higher gain across numbers of parents, but at the 60-year timepoint schemes with relatively more crosses and fewer progeny had higher gain. However, even at the 60-year timepoint, the optimal number of crosses was much less than the possible half-diallel of unique crosses.

Given the genetic parameters specified for the cassava program, the use of ∼8–16 parents, ∼24 crosses and ∼68 progeny per cross in each crossing block per year was the optimal distribution to maximize genetic gain at the 20-year time horizon (Figure 7A). At the 60-year time horizon, the optimal distribution was 16–32 parents, 60 crosses, and ∼30 progeny per cross (Figure 7B). To consider both short- and long-term interests of the breeding program, we chose to recommend use of 15–30 parents recycled from the combined PYT and AYT stages with 40 crosses and 40 progeny, given the constraint of the program to handle ∼1,632 materials to start.

### Improving the Process

The *improvement* step in the six-sigma method was adapted to breeding scheme improvement by using management tools like the A3 format to reflect the current and future state of the CES decision (subprocess) together with an action plan laying with detail the actions required to achieve the future state (Supplementary File 1; Anderson et al., 2011). We included a RACI chart (responsible, accountable, consulted and informed people in the improvement plan) to formalize the process to achieve the desired improvement. Is important to notice that a RACI chart can and should be employed during the management of the different tasks of the breeding process and not only for the continuous improvement of breeding schemes. We propose that the future state and actions included in the improvement plan should be guided by sound quantitative genetics principles and recommendations coming from state-of-the-art tools, such as evaluation of new strategies by genetic simulation (Mi et al., 2014; Gaynor et al., 2017; Pook et al., 2020, 2021). We expect that results obtained through simulation can identify close-to-optimal solutions and changes to the breeding CES tasks and decisions.

Based on the simulation findings, a meeting with the IITA-cassava breeding team was held to discuss the optimal scenarios revealed by simulations and the next steps. The recommendation to use between 15 and 30 parents in the crossing block depending on the target breeding-time-horizon was and to use a mixed crossing block of parents from both the PYT and the AYT was accepted by the team. The improvement plan developed by the IITA-cassava program included detailing the current and future state can be found in the Supplementary File 1. The improvement plan developed included actions like team agreement on the modification of the number of parents, number of crosses and number of progeny per cross used in the crossing block, the development of a new SOPs for the crossing block stage, training the technicians to execute the new SOPs, monitoring the genetic gain across years to confirm the positive change, among others.

### Controlling the Improvement Process

The *control* step was adapted to breeding program improvement by adding a monitoring section to the improvement plan that keeps track of the progress of the action plan through the inclusion of key performance indicators (KPIs), deadlines, and risks, as it does in other industrial processes. To monitor or control the progress of the improvement plan in the IITA-cassava, deadlines and key performance indicators for the different actions were defined and monitored to ensure that changes occur. Once the new process was adopted, we moved to the next possible crossing evaluation or selection decision that could be causing low rates of genetic gain. This process is still undergoing together with other improvements identified.

## Conclusion

There is tremendous potential of systematizing breeding as an industrial process and enabling continuous improvement methodologies (e.g., six-sigma) to the different crossing, evaluation, selection decisions and other parts of the breeding process. Successful implementation of these methodologies has potential to increase the rate of genetic gain and delivery of better products in breeding programs. To guarantee such improvements in genetic gain, the recommended changes must be near-optimal or at least better than the current strategy. We propose the use of genetic simulation to identify these solutions to guide the continuous improvement steps. The work with the IITA-cassava program resulted in improved resource allocations and adjustments to the proper number of parents to sustain gains for the breeding time horizon of interest. These and other improvements achieved through the same approach in other CES decisions are ongoing. We expect that this generalized framework will assist plant breeding professionals in transitioning toward conducting breeding as an industrial process, help prevent discontinuity and inconsistency in breeding pipelines and their schemes and implement a culture of continuous improvement in all areas of their breeding programs.

## Data Availability Statement

The datasets presented in this study can be found in online repositories. The names of the repository/repositories and accession number(s) can be found in the article/Supplementary Material.

## Author Contributions

GC-P, PC, JD, and MQ conceived the study. GC-P, ZG, and SS developed the software. DG, CW, and ML performed the simulations and applied the continuous improvement methodologies. IR, SK, EP, EK, EM, AA, and PK produced the data and ran the programs used for the study. All authors contributed to writing the manuscript and agreed to be accountable for the content of the work.

## Conflict of Interest

The authors declare that the research was conducted in the absence of any commercial or financial relationships that could be construed as a potential conflict of interest.

## Publisher’s Note

All claims expressed in this article are solely those of the authors and do not necessarily represent those of their affiliated organizations, or those of the publisher, the editors and the reviewers. Any product that may be evaluated in this article, or claim that may be made by its manufacturer, is not guaranteed or endorsed by the publisher.

## Footnotes

1 Breeding Pipeline (2021). *Breeding Pipeline**: Scope and Approach*. Available online at: https://globalrust.org/dggw/breeding-pipeline

2
https://www.cgiar.org/wp/wp-content/uploads/2018/05/SC6-04_Multi-Funder-Breeding-Initiative-update.pdf
